# Cholesterol lowering effect of a soy drink enriched with plant sterols in a French population with moderate hypercholesterolemia

**DOI:** 10.1186/1476-511X-7-35

**Published:** 2008-10-06

**Authors:** Christina Weidner, Michel Krempf, Jean-Marie Bard, Murielle Cazaubiel, Doris Bell

**Affiliations:** 1 Cognis GmbH, Monheim, Germany; 2 CHU Hôtel Dieu, Endocrinology Dept., Nantes, France; 3 Pharmacy Faculty, Nantes, France; 4 Biofortis, Nantes, France

## Abstract

**Background:**

Plant sterols are an established non-pharmacological means to reduce total and LDL blood cholesterol concentrations and are therefore recommended for cholesterol management by worldwide-renown health care institutions. Their efficacy has been proven in many types of foods with the majority of trials conducted in spreads or dairy products. As an alternative to dairy products, soy based foods are common throughout the world. Yet, there is little evidence supporting the efficacy of plant sterols in soy-based foods. The objective of this study was to investigate the effect of a soy drink enriched with plant sterols on blood lipid profiles in moderately hypercholesterolemic subjects.

**Methods:**

In a randomized, placebo-controlled double-blind mono-centric study, 50 subjects were assigned to 200 ml of soy drink either enriched with 2.6 g plant sterol esters (1.6 g/d free plant sterol equivalents) or without plant sterols (control) for 8 weeks. Subjects were instructed to maintain stable diet pattern and physical activity. Plasma concentrations of lipids were measured at initial visit, after 4 weeks and after 8 weeks. The primary measurement was the change in LDL cholesterol (LDL-C). Secondary measurements were changes in total cholesterol (TC), non-HDL cholesterol (non-HDL-C), HDL cholesterol (HDL-C) and triglycerides.

**Results:**

Regular consumption of the soy drink enriched with plant sterols for 8 weeks significantly reduced LDL- C by 0.29 mmol/l or 7% compared to baseline (p < 0.05). TC and non-HDL-C concentrations decreased by 0.26 mmol/l and 0.31 mmol/l (each p < 0.05), respectively. Mean reductions in total, LDL and non-HDL cholesterol were significantly greater than in the placebo group (p < 0.05). HDL-C and triglycerides were not affected. Compliance was very high (>96%), and products were well tolerated.

**Conclusion:**

Daily consumption of a plant sterol-enriched soy drink significantly decreased total, non-HDL and LDL cholesterol and is therefore an interesting and convenient aid in managing mild to moderate hypercholesterolemia.

## Background

Elevated blood LDL-cholesterol (LDL-C) concentrations are one of the major independent risk factors for cardiovascular diseases. Therapeutic lifestyle changes including dietary interventions, in particular a reduction of saturated fat and cholesterol, are established as first line therapy to reduce LDL-C. Additionally, plant sterols are recommended as a further dietary option to decrease LDL-C [1]. A multitude of clinical trials has documented that the daily consumption of 2 g plant sterols results in a LDL-C reduction by about 10% [2] which is additive to the cholesterol-lowering effect of a low-fat diet [3]. Plant sterols are naturally occurring compounds found in plant cells walls of several foods such as nuts, grains, vegetables and seeds. Their chemical structure is similar to that of cholesterol differing only in the presence of an additional methyl or ethyl group at carbon C-24. Plant sterols function as cholesterol-lowering ingredients, primarily by competing with cholesterol for solubilization into mixed micelles, and thus reduce intestinal absorption of dietary and biliary cholesterol [4].

Traditionally, plant sterols have been incorporated into high-fat products such as spreads and margarines. Recently, they have also been proven to be effective in low-fat foods such as low-fat yoghurts [5,6], milk [7,8] and yoghurt drinks [9,10]. However, only few studies have been performed on non-milk-based beverages [11,12] and, to the best we know, there is no published trial investigating the effectiveness of plant sterols incorporated into a soy-based beverage.

Soy-based products may present an appropriate food matrix for plant sterols since there is increasing evidence for the cardiovascular benefits of soy. Epidemiological data have indicated that Asian populations consuming soy as a dietary staple have a lower risk for cardiovascular diseases than those consuming a Western diet [13,14]. Moreover, some clinical trials investigating the effect of soy protein as part of a low-fat diet displayed significant LDL-C reductions [15]. Hence, in addition to already available milk-based products enriched with plant sterols, soy based foods with plant sterols may serve as another good option for people with hypercholesterolemia since it combines heart health benefits from both soy protein and plant sterols.

The aim of the following study was to demonstrate the cholesterol-lowering effect of 2.6 g/d of plant sterol esters (1.6 g/d of free plant sterol equivalents) incorporated into a soy drink in comparison to a control soy drink in a French population.

## Results

### Study subjects

A total of 50 subjects (19 men, 31 women) aged between 19 and 65 years (mean 40.36 ± 13.47 years) with known but untreated moderate hypercholesterolemia were randomly assigned to two groups. One subject withdrew from the study due to personal reasons after 4 weeks of intervention. Baseline characteristics of the study subjects were shown in Table 1. No significant differences were detected between groups. The products were overall well tolerated with no differences in reported adverse events between the study arms. With regard to subjects' compliance, the control and fortified soy drinks were consumed in similar manner and according to the recommendation (mean >96%).

**Table 1:** Characteristics of the subjects at baseline^1^

Variable	Control soy drink (n = 25)	Soy drink enriched with plant sterols (n = 25)	p
BMI (kg/m^2^)	23.94 ± 3.45	24.32 ± 3.10	0.2935
Systolic blood pressure(mmHg)	122.84 ± 10.64	120.76 ± 10.97	0.3591
Diastolic blood pressure(mmHg)	75.12 ± 8.09	78.04 ± 7.81	0.1699
Total cholesterol (mmol/l)	6.24 ± 0.77	6.42 ± 0.68	0.1429
Non-HDL-C (mmol/l)	4.67 ± 0.20	4.81 ± 0.57	0.2020
LDL-C (mmol/l)	4.10 ± 0.64	4.21 ± 0.52	0.1343
HDL-C (mmol/l)	1.57 ± 0.33	1.61 ± 0.38	0.3891

^1 ^All values are means ± SD

### Blood lipids

**Figure 1:**
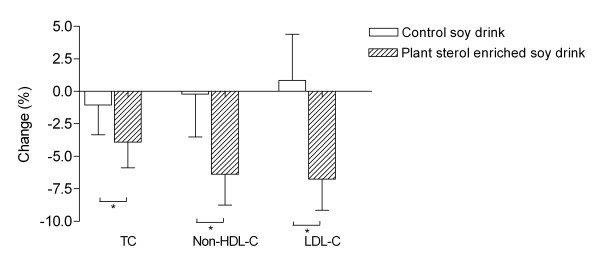
**Blood lipid changes (mean in % ± SE) between baseline (V0) and week 8 (V3)**. TC: Total cholesterol, Non-HDL-C: Non-HDL cholesterol, LDL-C: LDL cholesterol, p for inter group variation, *p < 0.05 vs. control.

Serum lipid concentrations are presented in Table 2. At week 4, a significant reduction of LDL-C by 0.32 mmol/l or 7.15% compared to baseline (p < 0.01) was observed in the plant sterol group. At week 8, LDL-C was decreased by 0.29 mmol/l corresponding to a relative reduction of 6.76% (p < 0.01). Total cholesterol was diminished by 0.32 mmol/l (p < 0.01) and 0.26 mmol/l (p < 0.01) from baseline at week 4 and 8, respectively. In addition, non-HDL-cholesterol was reduced by 0.35 mmol/l (p < 0.01) and 0.31 mmol/l (<0.01). HDL-C and triglycerides were not affected. The control group did not show any significant modifications of the blood lipid profile over the course of the treatment period. Comparing the percentage changes of blood lipids after 8 weeks between verum and placebo group, a significant difference was observed for total (p < 0.05), LDL (p < 0.05), and non-HDL-C (p < 0.05) (Figure 1). There were no clinically relevant changes in any other laboratory values.

**Table 2:** Serum lipid concentrations at baseline and at week 4 and week 8^1^

	Control soy drink	Soy drink enriched with plant sterols
Total cholesterol (mmol/l)		
Baseline (V0)	6.24 ± 0.77	6.42 ± 0.68
Week 4 (V2)	6.12 ± 0.64	6.10 ± 0.64^a^
Week 8 (V3)	6.16 ± 0.79	6.16 ± 0.79^a^
Change (%) between V2 and V0	-1.34	-4.76
Change (%) between V3 and V0	-1.05	-3.91^b^

LDL-cholesterol (mmol/l)		
Baseline (V0)	4.10 ± 0.64	4.21 ± 0.64
Week 4 (V2)	4.00 ± 0.74	3.89 ± 0.44^a^
Week 8 (V3)	4.09 ± 0.73	3.92 ± 0.61^a^
Change (%) between V2 and V0	-1.91	-7.15
Change (%) between V3 and V0	+0.84	-6.76^b^

Non-HDL-cholesterol (mmol/l)		
Baseline (V0)	4.67 ± 0.70	4.81 ± 0.57
Week 4 (V2)	4.58 ± 0.76	4.46 ± 0.56^a^
Week 8 (V3)	4.61 ± 0.78	4.50 ± 0.76^a^
Change (%) between V2 and V0	-1.31	-6.95^b^
Change (%) between V3 and V0	-0.22	-6.38^b^

HDL-cholesterol (mmol/l)		
Baseline (V0)	1.57 ± 0.33	1.61 ± 0.38
Week 4 (V2)	1.55 ± 0.35	1.64 ± 0.44
Week 8 (V3)	1.53 ± 0.35	1.66 ± 0.45
Change (%) between V2 and V0	-0.52	+1.92
Change (%) between V3 and V0	-1.69	+3.07

Triglyceride (mmol/l)		
Baseline (V0)	1.23 ± 1.29	1.29 ± 0.48
Week 4 (V2)	1.25 ± 0.73	1.24 ± 0.57
Week 8 (V3)	1.12 ± 0.49	1.27 ± 0.62
Change (%) between V2 and V0	+0.76	-2.33
Change (%) between V3 and V0	-5.35	+0.09

^1 ^All values are mean ± SD.

^a ^significant compared to baseline, p < 0.05.

^b ^significant compared to control, p < 0.05.

### Satisfaction questionnaire

With regard to product satisfaction, estimated by the volunteers completing questionnaires at the end of the study, no difference was apparent between the control product and the plant sterol enriched product. The taste was deemed to be acceptable (data not shown) and a tendency to consume this type of product preferably in the morning was confirmed (Table 3). As to the habitual consumption, 24% of the verum group and 8% of the control group stated to customary consume anti-cholesterol products, whereas the 76% and 92%, respectively did not regularly use cholesterol-lowering products.

**Table 3:** Satisfaction questionnaire: When would you consume the product? Multiple answers possible

% responses	Control soy drink	Soy drink enriched with plant sterols
At breakfast	67	64
As a snack during the morning	56	54
During the meal (lunch or dinner)	4	8
During the afternoon	8	17
As a snack during the afternoon	20	25
In the evening, after dinner	0	4
Just before going to bed	4	0
Don't know	4	4

## Discussion

Dietary intervention is the cornerstone of non-pharmacological strategies to reduce LDL-C and thereby improving cardiovascular health. Regular consumption of plant sterol-enriched foods and an increase in dietary fiber intake are recommended to enhance the cholesterol-lowering dietary modifications [1]. In the present controlled, double-blind trial, a significant reduction in total, non-HDL- and LDL-cholesterol was observed after daily consumption of a plant sterol-enriched soy beverage. The magnitude of the LDL-C reduction in this trial (7%) is in accordance with previous reported studies in which LDL-C was decreased by at least 5% compared to control. For instance, in adults with mild hypercholesterolemia, consumption of plant sterols provided as spreads reduced LDL-C by 5.4% [16]. The intake of plant sterol-enriched milk and yoghurt resulted in LDL-C decreases by 7.1% [17] and 6%, respectively [18].

Traditionally, high-fat products such as spreads were chosen as food matrix for plant sterols due to the fat-soluble nature of plant sterol esters. A meta-analysis of randomized, placebo-controlled trials using fatty food matrix enriched with sterols reported mean LDL-C reductions ranging from 6.7% to 11.3% according to dose [2]. More recent research has focussed on the cholesterol-lowering effects of plant sterol-enriched low-fat foods, such as yoghurts, yoghurt drinks, milk and orange juice. As individuals with elevated LDL-C concentrations are advised to limit their dietary saturated fat intake, these products may be of particular interest for the management of hypercholesterolemia. Soy products such as the soy drink used in this trial are known to be low in saturated fat and might therefore be an appropriate matrix for plant sterols as well.

However, the effects of plant sterol-enriched low-fat foods on cholesterols are inconsistent in published studies. One study, such as Jones et al. [19], failed to show a significant improvement of the blood lipid profile after intake of plant sterols provided as low-fat and non-fat beverages compared to control, whereas other studies reported that plant sterols were effective in reducing LDL-C when incorporated into low-fat or non-fat foods [1,5,11,17,20] or even when provided as capsules [21,22]. The degree of solubilization and dispersion of plant sterols as well as the intake occasion appears to account in part for the variable results [23]. Doornbos et al. [9] demonstrated that a low-fat single dose drink significantly lowered LDL-C both when taken with or without a meal. However, a substantially larger decrease in blood cholesterol concentrations was achieved when a single-dose drink was consumed with or directly after lunch (-9.3% vs. – 5.1%, p < 0.005). As plant sterols reduce the intestinal cholesterol absorption by competing with cholesterol for micellar solubilization, it seems plausible that factors affecting gastrointestinal transit time and stimulating bile flow may influence the cholesterol-lowering efficacy of plant sterols. The soy juice consumed in this study was taken with breakfast or during the morning. As the breakfast was not standardized and the majority of participants had a rather small than a comprehensive breakfast according to French consumption habits, it cannot be ruled out that these aspects had an impact on the cholesterol-lowering efficacy of the plant sterol-enriched soy drink. Thus, the intake of the soy drink with lunch or dinner might have resulted in greater cholesterol-lowering effects.

Plant sterols are recommended in addition to dietary changes for successful non-pharmacological intervention of elevated cholesterol. Moreover, the American Heart Association proposes the inclusion of at least 25 g/d of soy protein in a low fat, low cholesterol diet to reduce the risk of coronary heart disease. Clinical trials have reported that a daily consumption of 25–50 g/d of soy protein decreases LDL-C by about 4 to 8% [24], for instance. While plant sterols reduce the intestinal absorption of cholesterol, soy protein is thought to lower blood cholesterol concentrations via a decrease in hepatic cholesterol synthesis [25] or through an increase of plasma cholesterol clearance [26]. Therefore, it may be possible that a combination of these natural ingredients has additive effects and may therefore modify the blood lipid profile more favourably than a single ingredient. However, very few studies have investigated a possible synergistic effect yet [27-29].

In the present study, the control soy drink did not significantly affect blood lipids over 8 week period, which could be due to the low soy protein content (7 g/d). In contrast, the soy drink enriched with plant sterols significantly decreased LDL-C by 7% suggesting a beneficial effect from plant sterols.

Albeit low-dose soy protein products alone may not allow individuals with elevated cholesterol levels to achieve there lipid targets, they are nonetheless a meaningful matrix for the incorporation of plant sterols, since soy products do not unbalance the daily energy intake or modify the pattern of dietary habits. The latter aspect may be important to obtain a good patient compliance during long-term intervention [27]. Moreover, the substitution of soy for animal protein can result in lower saturated fat and cholesterol intakes, thereby indirectly leading to a more favourable blood cholesterol level and potentially reducing the risk of coronary heart disease [30].

As to the satisfaction of volunteers with the product taste, no difference was observed between treatment and control beverage indicating a proper introduction of the plant sterols into the soy drink. The majority of subjects preferred to consume of the cholesterol-lowering drink at breakfast or as a snack during the morning or afternoon. Since studies have shown plant sterols to be more effective when consumed in combination with a (main) meal [9], consumption recommendations on the package of the product may be helpful to ensure a maximum effect for the consumers. The fact that a large part of subjects did no habitually consume anti-cholesterol products such as plant sterols suggests that people having elevated cholesterol levels are often not familiar with or not aware of such cholesterol-lowering options. Consequently, intensified public information about non-pharmacological interventions for cholesterol management is desirable and may help to reduce the high prevalence of hypercholesterolemia.

## Conclusion

A soy drink enriched with plant sterols is effective in lowering total and LDL cholesterol in subjects with moderate hypercholesterolemia. Therefore, it may be used as another potential dietary approach to the management of hypercholesterolemia. A replacement of animal-based products by soy-based product may further contribute to a reduction of blood cholesterol levels. Moreover, soy protein is proposed to have hypocholesterolemic properties itself. Whether the effects of plant sterols and soy protein are synergistic remains to be further investigated in future clinical trials.

## Methods

The study was performed according to Good Clinical Practice (GCP) at Biofortis, Nantes, France and was approved by the Ethics Committee of the Comité de Protection des Personnes se Prêtant à la Recherche Biomedicale (CCPPRB) des Pays de la Loire, Nantes, France. Subjects were recruited via newspaper advertisement or have been selected from the volunteers' base of the study center.

### Subjects

Subjects with known but untreated moderate hypercholesterolemia were screened on the basis of following inclusion criteria: LDL-C 130–220 mg/dl depending on other cardiovascular risk factors present (as to the AFSSAPS criteria, the inclusion LDL-C limit for subjects without any other risk factors was 220 mg/dl, for subjects with one additional risk factor 190 mg/dl, and for those with two additional risk factors 160 mg/dl); body mass index ≤ 30 kg/m^2 ^triglyceride concentrations < 200 mg/dl; no severe disease necessitating pharmacological intervention; no hepatic disease, gastrointestinal complaints, no thyroid dysfunction or any kidney disease; no personal history of cardiovascular illness; no diabetes; no hypertension in terms of the WHO criteria (140/90 mmHg) and no known food allergy.

### Experimental design

**Figure 2:**
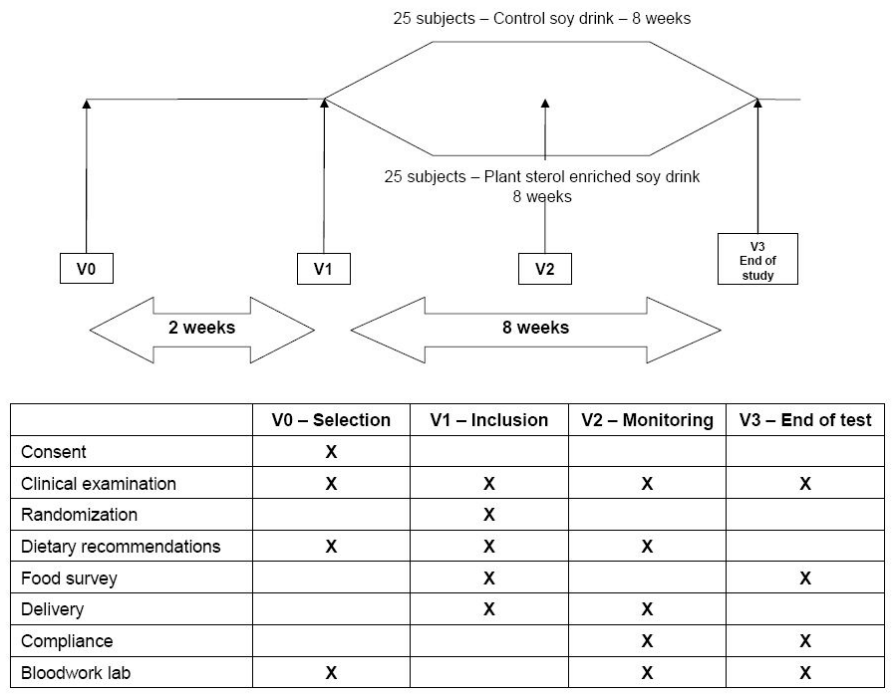
Study program.

The study was a mono-centric, randomized, double blind, placebo-controlled trial in parallel groups of 25 subjects each. All subjects gave informed consent. The study was conducted over a 10-week period including a 2-week run-in phase (general dietary recommendations, exclusion of plant sterol enriched foods) and 8 weeks of intervention (Figure 2). After run-in, subjects were randomized to either 200 ml soy drink without plant sterols (control) or 200 ml soy drink enriched with plant sterols (Vegapure 95E^®^, Cognis GmbH, Monheim, Germany). The soy beverages were consumed either with breakfast or during the morning before noon. Participants were encouraged to continue with their normal diet and exercise pattern during the study period.

Clinical examination was performed at selection (visit 0, V0), inclusion (V1), follow-up (V2, week 4) and end of intervention (V3, week 8). Blood samples were taken at V0, V2, and V3, dietary and lifestyle advice was given at V0, V1, and V2. A food survey was performed at V1 and V3. The compliance with the product was established by counting the empty packages on V2 and V3. Adverse events were reported by the volunteers by filling in a questionnaire at V1, V2, and V3. During visit V3, the volunteers filled in a questionnaire regarding the overall satisfaction with the product.

### Products

The control product was 200 ml of soy drink (Nutrition & Santé, Revel, France) without plant sterols. The test product was the same soy drink enriched with 2.6 g/d plant sterol esters (1.6 g/d of free plant sterol equivalents, Vegapure^®^95E, Cognis GmbH, Monheim, Germany). For the product formulation and composition of plant sterols see Tables 4 and 5.

**Table 4:** Macronutrient composition of the soy drink products used in the study

Average content of 100 ml soy drink	Control soy drink	Soy drink enriched with plant sterols
Calories (kcal)	51.1	65
Energy (kj)	214.3	271
Protein (g)	3.5	3.6
Carbohydrates (g)	4.1	4.3
Of which sugars	2.3	3.3
Fat (g)	2.3	3.7
Of which free sterol	0	0.8
Fibres (g)	0.47	0.49
Sodium (g)	0.1	0.1

**Table 5:** Sterol composition of Vegapure 95E^®^

Sterol composition (as free sterols)	By GC, rel. area (%), typical values (max.*)
Beta-Sitosterol	69.1% (max. 80%)
Beta-Sitostanol	7.9% (max.15%)
Campesterol	15.3% (max. 40%)
Campestanol	1.0% (max. 5%)
Stigmasterol	0.8% (max. 30%)
Brassicasterol	2.8% (max. 3%)
Other sterols/stanols	2.7% (max. 3%)

* according to European Commission Decision

### Outcomes

The primary outcome was LDL cholesterol. Secondary outcomes were total cholesterol, non-HDL cholesterol, HDL cholesterol and triglycerides. Blood samples were taken after a 12-h overnight fast. LDL cholesterol calculation was performed using Friedewald equation.

Additionally, the individual feedback with regard to product taste and consumption habits was documented.

### Statistical analysis

SAS software version 8.2 was used for the statistical analysis. The main effective criterion was the change in the concentration of serum LDL-cholesterol (LDL-C). The study was designed to detect a difference in LDL-C by 8% between groups with an alpha risk of 0.05, and a beta risk of 0.01 or 0.02. The effect of the treatment was evaluated by non-parametric Wilcoxon test which compared the data obtained after four and eight weeks of treatment in the group receiving the control soy drink with those obtained in the group receiving plant sterol enriched soy drink. The same analysis was performed for comparisons of intra-group changes (V0 vs. V2, V0 vs. V3). The analysis was conducted according to "intention to treat" population comprising all the subjects included in the study. For the subject leaving the study prematurely, a blood sample was taken at time of his premature departure.

The discontinuous data of the satisfaction questionnaire were analyzed by means of a chi^2 ^test and the questions to which a mark was attached were analyzed by a Wilcoxon test, after calculating the mean values obtained from each group.

## List of abbreviations

AFSSAPS: Agence Française de Securite Sanitaire des Produits de Sante; CCPPRB: Comité de Protection des Personnes se Prêtant à la Recherche Biomedicale; d: day; g: gram; GCP: good clinical practice; h: hours; HDL-C: high density lipoprotein cholesterol; kg: kilogram; LDL-C: low density lipoprotein cholesterol; ml: millilitre; mmol: millimole; non-HDL-C: non-high density lipoprotein cholesterol; V: visit; vs.: versus.

## Competing interests

MK, JMB and MC declare that they have no competing interests. MC is clinical study manager at Biofortis, France. CW and DB are research scientists at Cognis GmbH, Germany who were involved in interpreting the data and preparing the manuscript, but were not involved in conducting the study.

## Authors' contributions

All authors have read and approved the final manuscript. MK, JMB and MC contributed to the protocol writing. MC and Biofortis contributed to the volunteer recruitment. MC, JMB and Biofortis contributed to the data analysis and result interpretation. CW wrote the manuscript, DB revised the manuscript. All authors have read and approved this manuscript.
